# Supplementary material for: Impairments of spatial memory in an Alzheimer’s disease model via degeneration of hippocampal cholinergic synapses
Source: Nat Commun. 2017 Nov 22;8:1676. doi: 10.1038/s41467-017-01943-0 (PMC5698429; doi:10.1038/s41467-017-01943-0)
Supplement: Supplementary file 1 — Supplementary Information [file 41467_2017_1943_MOESM1_ESM.pdf]

Supplementary Table 1: A list of the mutant lines of mice

Tg1	Tg2	Tg1+Tg2	Experiments
**ChATs-Cre$^{GFP+/+}$**			**Hsv-DIO-mCherry virus traces vChATs innervating dNGIs**
**Nestin-Cre^{ER}**	**TVA/G$^{loxp/loxp}$**	**dNGIs$^{TVA/G+/+}$**	**Rabies-mCherry virus traces dNGIs presynaptic vChATs**
**ChATs-Cre$^{GFP+/+}$**			**Hsv-DIO-mCherry virus labels vChATs innervating dNGIs**
**ChATs-Cre$^{GFP+/+}$**			**Hsv-DIO-mCherry virus labels vChATs innervating dNGIs**
**ChATs-Cre$^{GFP+/+}$**		**dNGIs$^{M1siRNA+}$**	**rAVV1/2-M1siRNA virus expresses M1siRNA in dNGIs**
**AD mice**	**vChATs^{ChR2+}**	**AD/vChATs^{ChR2+}**	**HSV-DIO-mCherry virus labels vChATs innervating dNGIs in AD mice**
**AD mice**	**vChATs^{ChR2+}**	**AD/dNGIs$^{M1siRNA+}$**	**rAVV1/2-M1siRNA virus expresses M1siRNA in dNGIs of AD mice**

Note: Tg1 indicates transgenic 1 mice; Tg2 indicates transgenic 2 mice. Tg1+Tg2 indicates breeding of transgenic 1 and transgenic 2 mice. In the present study, AD mice were crossed with vChATsChR2+ mice, resulting in both AD/vChATsChR2+ and the non-transgenic control/vChATsChR2+ mice and control/vChATsChR2+ from the same litter of AD/vChATsChR2+ mice were used as the controls.

Supplementary Table 2. The stastical analysis results for all figures

	Test Used	n		Descriptive STATS (Average, Variance)	P Value	Degrees of Freedom & F/t/z/R/etc Value
Figure Number	Which Test	Exact Value	Defined?	Reported?	Exact Value	Value
2d	two-way ANOVA with Bonferroni post-hoc test	28 cells per group	36 mice from 6 groups	error bars are mean SEM	1 mV, p=0.35; 2 mV, p=0.26; 3 mV, p=0.17; 4 mV, p=0.04; 5 mV, p=0.007	1 mV, $F_{5,164}$=1.63; 2 mV, $F_{5,164}$=1.85; 3 mV, $F_{5,164}$=2.02; 4 mV, $F_{5,164}$=2.76; 5 mV, $F_{5,164}$=3.30
3c	two-way ANOVA with Bonferroni post-hoc test	9 mice per group per time	216 mice from 4 groups	error bars are mean SEM	1 DAI, p=0.39; 3 DAI, p=0.42; 7 DAI, p=0.33; 11 DAI, p=0.26; 21 DAI, p=0.0009; 28 DAI, p=0.0005	1 DAI, $F_{3,213}$=1.52; 3 DAI, $F_{3,213}$=1.65; 7 DAI, $F_{3,213}$=1.83; 11 DAI, $F_{3,213}$=2.15; 21 DAI, $F_{3,213}$=4.15; 28 DAI, $F_{3,213}$=5.21
3e	two-way ANOVA with Bonferroni post-hoc test	11 mice per group	44 mice from 4 groups	error bars are mean SEM	p=0.0075	$F_{3, 41}$=4.85
3f	two-way ANOVA with Bonferroni post-hoc test	9 mice per group per time	216 mice from 4 groups	error bars are mean SEM	1 DAI, p=0.43; 3 DAI, p=0.38; 7 DAI, p=0.41; 11 DAI, p=0.32; 21 DAI, p=0.0082; 28 DAI, p=0.0008	1 DAI, $F_{3,213}$=1.49; 3 DAI, $F_{3,213}$=1.55; 7 DAI, $F_{3,213}$=1.92; 11 DAI, $F_{3,213}$=2.33; 21 DAI, $F_{3,213}$=4.29; 28 DAI, $F_{3,213}$=5.05
4b	repeated measures 2-way ANOVA	11 mice per group	44 mice from 4 groups	error bars are mean SEM	p=0.00082	$F_{1.43}$ = 11.02
5b	repeated measures 2-way ANOVA	11 mice per group	44 mice from 4 groups	error bars are mean SEM	p=0.00098	$F_{1.43}$ = 9.87
5e	two-way ANOVA with	11 mice per group	44 mice from 4 groups	error bars are mean SEM	Non-TBS, p = 0.0012; TBS, p =	$F_{1.43}$ = 9.79

	Bonferroni post-hoc test				0.00078	
6b	two-way ANOVA with Bonferroni post-hoc test	5 mice per group	20 mice from 4 groups	error bars are mean SEM	p = 0.0051	$F_{1.19}$ = 10.8
7a	two-way ANOVA with Bonferroni post-hoc test	9 mice per group	36 mice from 4 groups	error bars are mean SEM	p = 0.0019	$F_{1.35}$ = 10.91
7b	repeated measures 2-way ANOVA	11 mice per group	44 mice from 4 groups	error bars are mean SEM	p=0.00072	$F_{1.43}$ = 7.99
7c	two-way ANOVA with Bonferroni post-hoc test	9 mice per group	36 mice from 4 groups	error bars are mean SEM	p=0.00081	$F_{1.35}$ = 38.93
7d	repeated measures 2-way ANOVA	11 mice per group	44 mice from 4 groups	error bars are mean SEM	2-CSD, p=0.00097; BrdU, p=0.00026	2-CSD, $F_{1.43}$ = 35.07; BrdU, $F_{1.43}$ =68.61
S5b	two-way ANOVA with Bonferroni post-hoc test	9 mice per group	36 mice from 4 groups	error bars are mean SEM	p=0.0052	$F_{1.35}$ = 9.85
S6b	paired t-test	36 cells	18 mice from 2 groups	error bars are mean SEM	p=0.00046	t_{35}=6.53
S6c	two-way ANOVA with Bonferroni post-hoc test	9 mice per group	36 mice from 4 groups	error bars are mean SEM	p=0.00093	$F_{1.35}$ = 7.644
S10b	two-way ANOVA with Bonferroni	5 mice per group	20 mice from 4 groups	error bars are mean SEM	p = 0.00085	$F_{1.19}$ = 15.36

	post-hoc test					
S10d	two-way ANOVA with Bonferroni post-hoc test	9 mice per group	36 mice from 4 groups	error bars are mean SEM	p=0.00075	$F_{1.35} = 16.53$
S11b	two-way ANOVA with Bonferroni post-hoc test	9 mice per group	36 mice from 4 groups	error bars are mean SEM	p=0.0029	$F_{1.35} = 13.21$
S11g	repeated measures 2-way ANOVA	11 mice per group	44 mice from 4 groups	error bars are mean SEM	p=0.0039	$F_{1.43} = 11.89$
S12c	two-way ANOVA with Bonferroni post-hoc test	9 mice per group	36 mice from 4 groups	error bars are mean SEM	Non-TBS, p = 0.00082; TBS, p = 0.00091	$F_{1.35} = 7.22$
S14c	two-way ANOVA with Bonferroni post-hoc test	9 mice per group	36 mice from 4 groups	error bars are mean SEM	p=0.0018	$F_{1.35} = 29.01$
S14d	two-way ANOVA with Bonferroni post-hoc test	9 mice per group	36 mice from 4 groups	error bars are mean SEM	p=0.00068	$F_{1.35} = 35.25$
S14e	two-way ANOVA with Bonferroni post-hoc test	8 mice per group	32 mice from 4 groups	error bars are mean SEM	p=0.0012	$F_{1.31} = 8.91$

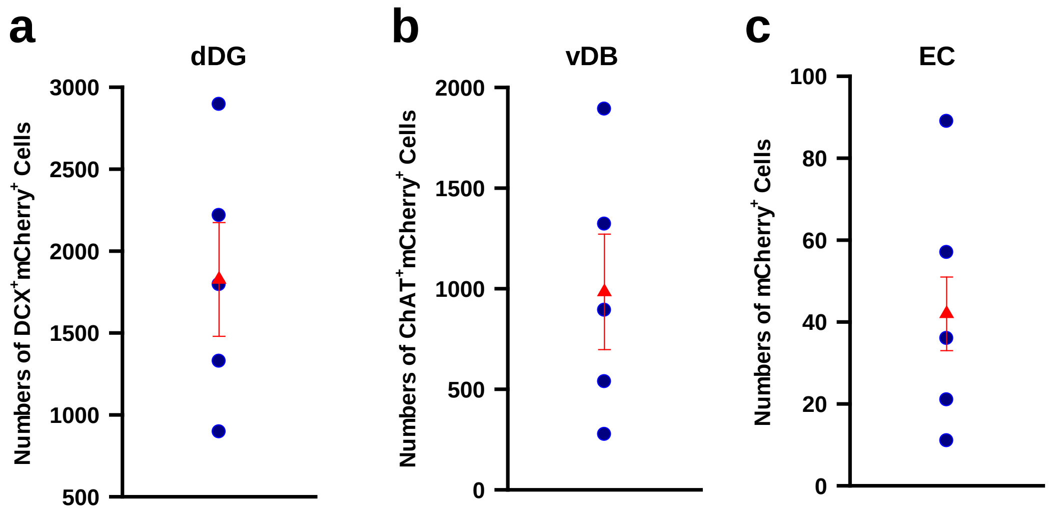

Supplementary Fig. 1. (**a**) The actual (blue circles) and the averaged (red triangles, mean ± SEM) numbers of $DCX^+mCherry^+$ cells in the dDG region of 5 individual mice from Fig. 1d. (**b**) The actual (blue circles) and the averaged (red triangles, mean ± SEM) numbers of $ChAT^+mCherry^+$ cells in the vDB region of 5 individual mice from Fig. 1h. (C) The actual numbers (blue circles) and the averaged (red triangles, mean ± SEM) numbers of $mCherry^+$ cells in the entorhinal cortical (EC) region of 5 individual mice from Fig. 1f.

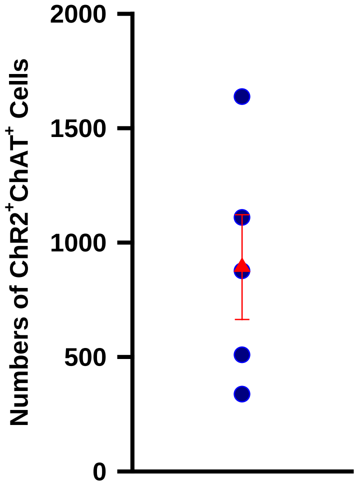

Supplementary Fig. 2. The actual numbers (blue circles) and mean ± SEM of $ChR2^{+}ChAT^{+}$ cells in the vDB region of 5 individual mice from Fig. 2d

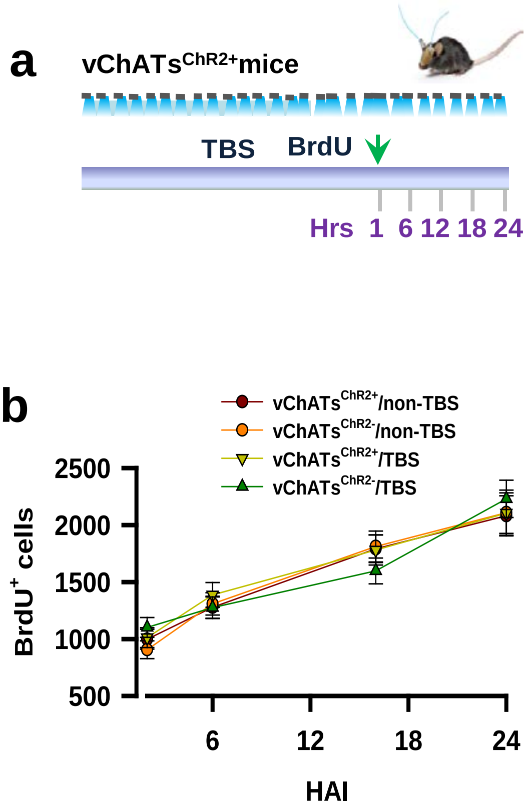

Supplementary Fig.3. **Activation of vChATs does not affect progenitor cell proliferation.** (**a**) Experimental schedule shows that adult mice at 120 ± 2 days old of age were applied with 16 consecutive days of TBS. One day before end of TBS application. A single dose of BrdU was administered. BrdU labeling was done in 1, 6, 12, 18 or 24 hrs after the BrdU injection (HAI). (**b**) A bar graph shows the numbers of BrdU^{+} cells in the dDG region of vChATs^{ChR2+} and vChATs^{ChR2-} mice treated without (non-TBS) or with TBS. Data are mean ± SEM (n = 9 mice/group).

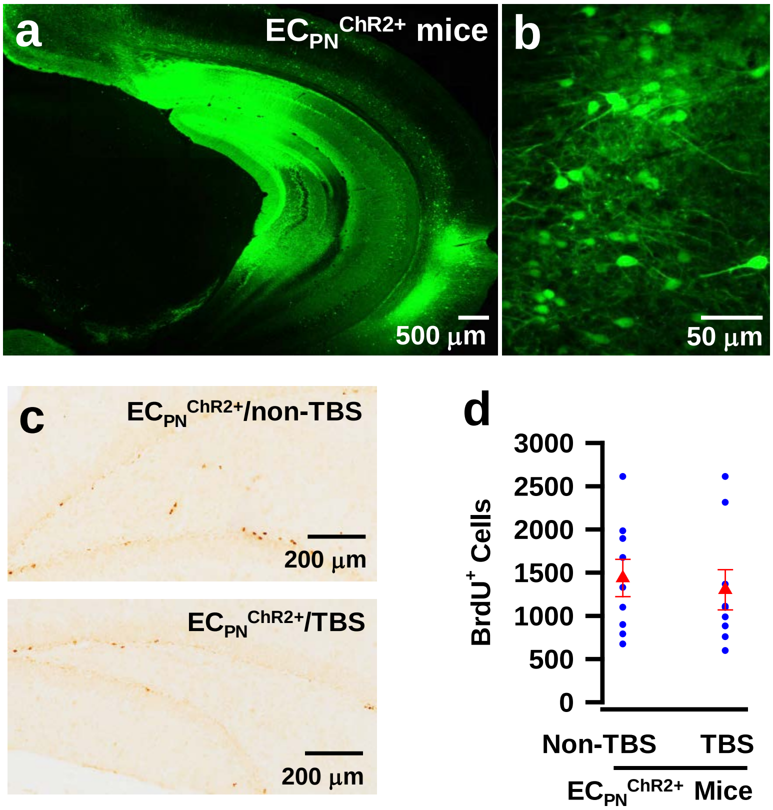

Supplementary Fig.4. (**a**) Generation of EC_{PN}^{ChR2+} mice by crossing ChR2-GFP$^{loxP/loxP}$ mice with CaMKIIα-Cre mice. (**b**) High magnification shows the expression of ChR2-GFP in excitatory pyramidal neurons in EC_{PN}^{CHR2+} mice. (**c**) EC_{PN}^{ChR2+} mice at 120 ± 2 days old of age were administered with a single dose of BrdU. 7 days after the administration, mice were applied without (non-TBS) or with 16-days TBS. 5 days after the end of TBS, mice were euthanized for BrdU labeling. (d) The actual (blue circles) and the averaged (red triangles, mean ± SEM) numbers of BrdU$^+$ cells in the dDG region of the individual EC_{PN}^{ChR2+} mice treated without (non-TBS) or with TBS (n = 9 mice per group, $F_{1.18}$ = 0.18, p = 0.67, *ANOVA*).

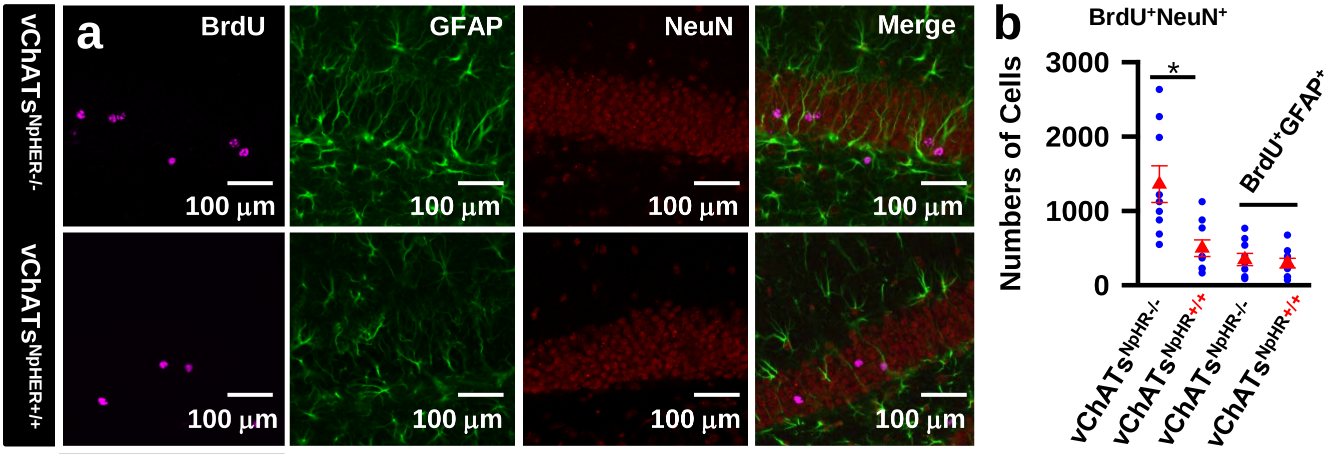

Supplementary Fig. 5. **Inhibition of vChATs reduces the number of new neurons without affecting the glia cell proliferation.** (**a**) Representative images show the labels of BrdU (pink), GFAP (green), and NeuN (red) and their merges in the dDG region of the vChATs^{NpHR+} and vChATs^{NpHR-} mice 5 days after 16-days ALL treatment. (**b**) The actual (blue circles) and the averaged (red triangles, mean ± SEM) numbers of BrdU^{+}NeuN^{+} and BrdU^{+}GFAP^{+} cells in the dDG region of the vChATs^{NpHR+} and vChATs^{NpHR-} mice 5 days after 16-days ALL treatment (n = 9 mice/group, *p*=0.0052, F1,35=9.85, two-way *ANOVA*).

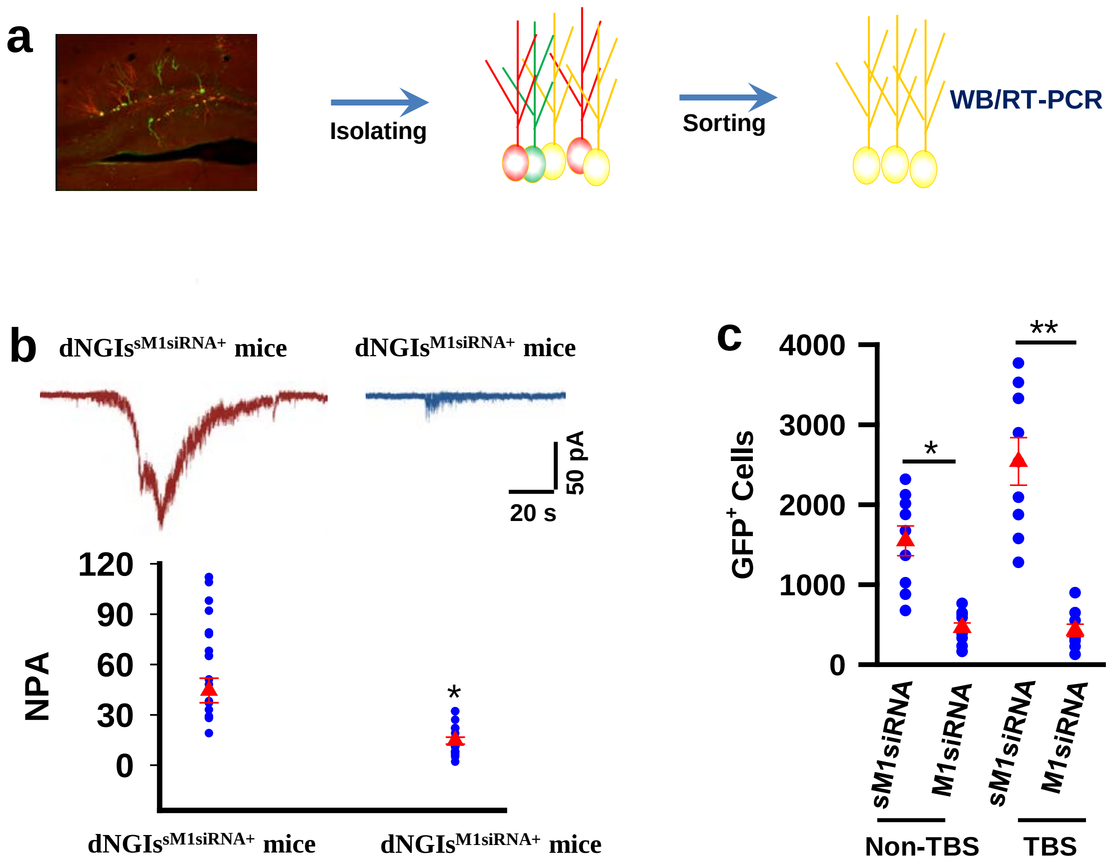

Supplementary Fig. 6. Expression of M1siRNA blocks cholinergic synaptic transmission in dNGIs and inhibits the dNGIs survival. (a) Illustration shows the experimental procedures of Western blots (WB) and RT-PCR analysis from isolated cells. (**b**) Representative recordings (top) and the normalized peak amplitude (NMP, bottom) of synaptic currents in dNGIs evoked by illuminating vChATs in the slices from both the dNGIs$^{M1siRNA+}$ mice at 120 days old of age and the age-matched dNGIs$^{sM1siRNA+}$ mice. Synaptic currents evoked by laser lights at an intensity of 5 mV were normalized to synaptic responses evoked at an intensity of 1 mV (defined as 1.0). A plot shows the actual (blues circles) and the averaged (red triangles, mean ± SEM) values of NPA from the individual mice (n = 18 cells/9 mice/group, *p* =0.00046, t_{35}=6.53; paired *t*-tests). (**c**) The actual (blue circles) and the averaged (red triangles, mean ± SEM) numbers of GFP^{+} cells in the dDG region from the individual the dNGIs$^{M1siRNA+}$ and dNGIs$^{sM1siRNA+}$ mice treated without (non-TBS) or with TBS are plotted (n = 9 mice/group, $F_{1.35}$ = 7.644, *P = 0.00093, **p = 0.00012, two-way *ANOVA*).

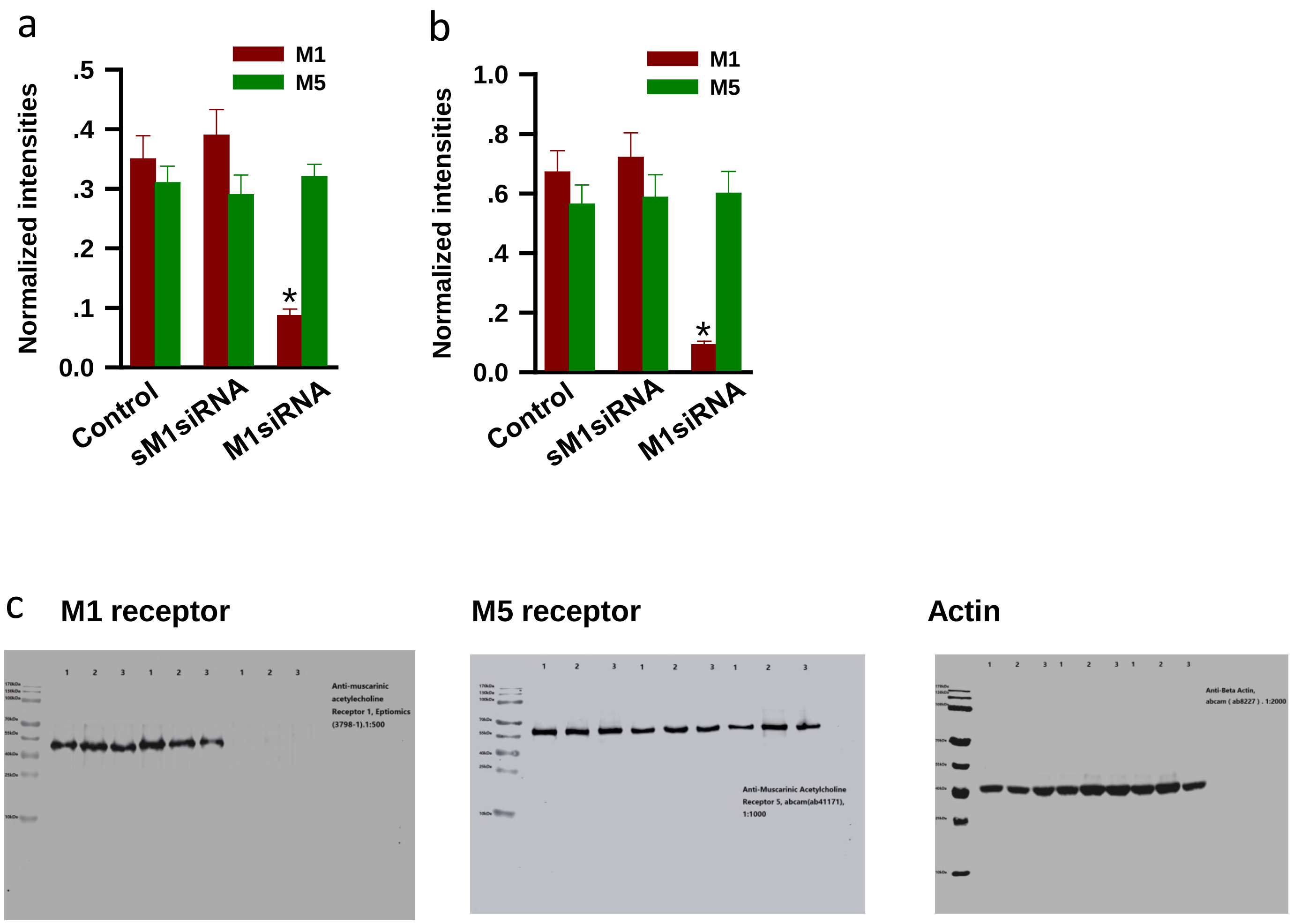

Supplementary Fig. 7. (a, b) Bar graphs show the normalized intensities of PCR (a) and Western blots (b) from Fig. 3g. The intensities of 3 replicates in each experimental condition were averaged. The averaged intensities were then normalized to the respective actin. Data are mean ± SEM (n = 4, *p < 0.001, compared to control, Student *t*-tests). (**c**) The original images of Western blots in Fig. 3g.

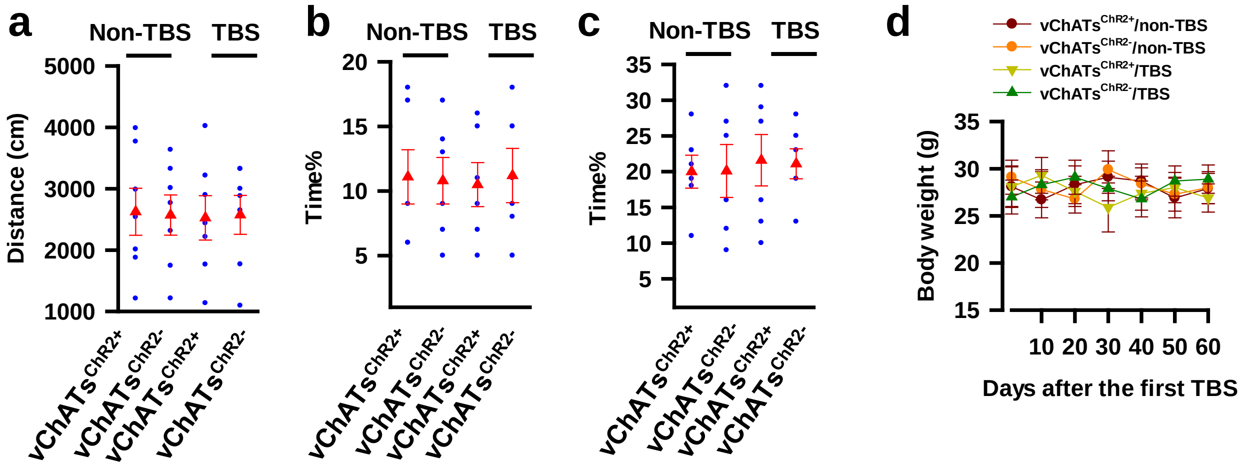

Supplementary Fig. 8. TBS does not affect the performance in the open field tests. (**a-c**) The actual (blue circles) and the averaged (red triangles, mean ± SEM) values of distance traveled (**a**), the percentage spent on familiar (**b**) and novel (**c**) objects in the open field are plotted (n = 7 mice/group). **(d)** Body weight was not changed throughout the period of TBS. Data are mean ± SEM (n = 7 mice/group).

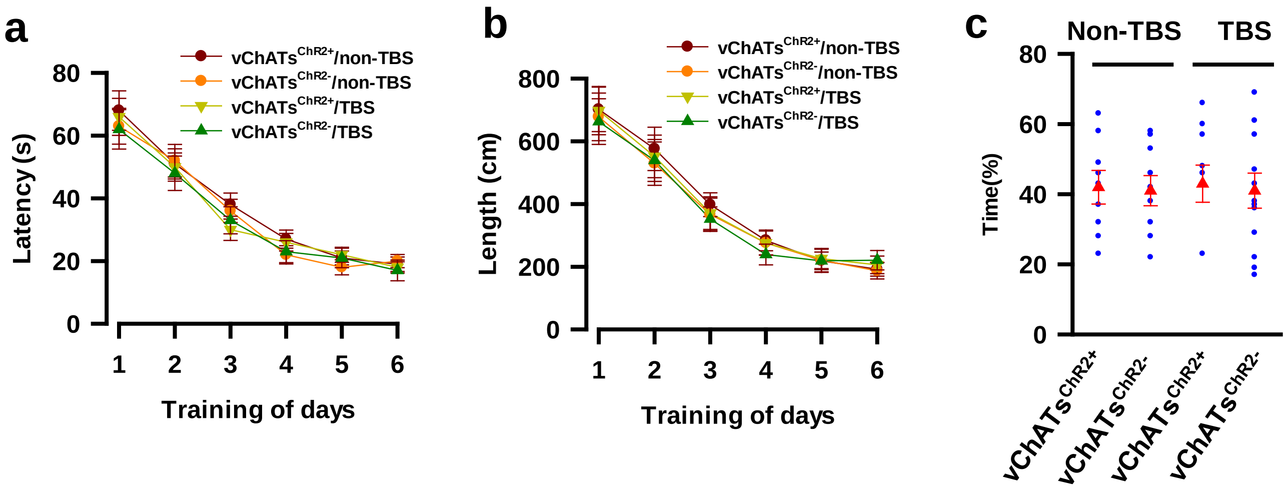

Supplementary Fig. 9. Activation of vChATs does not alter the performance in the Morris water maze tests (a-c). Latencies (**a**) and swim length (**b**) in searching for a hidden platform in the maze during the training session and the actual (blue circles) and the averaged (red triangles, mean ± SEM) values of the percentage time spent in a targeting quadrant during the probe trials (**c**) from the individual animals are plotted (n = 9 mice/group).

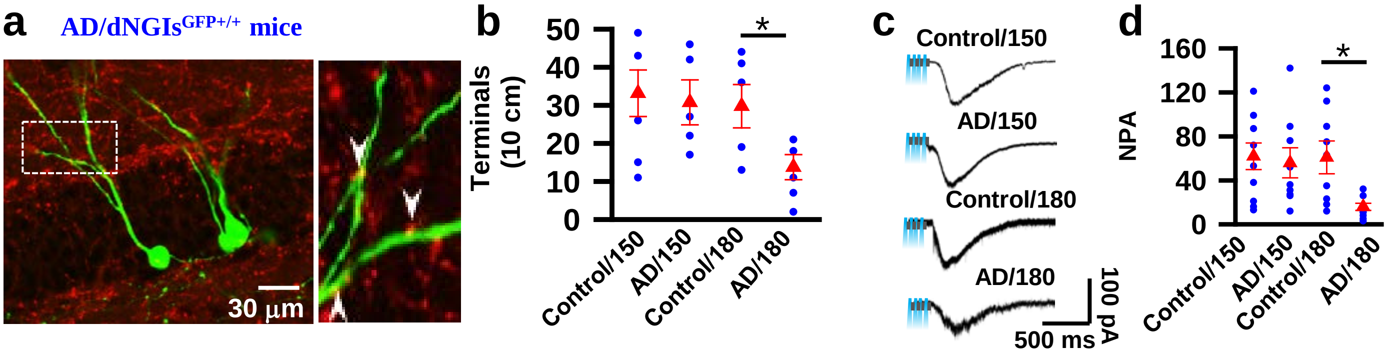

Supplementary Fig. 10. **Cholinergic synaptic transmission is impaired in AD mice. (a, b)** Representative images (**a**) of the ChATs-labeled terminals (red) targeting the individual dNGIs in AD/dNGIs$^{GFP+/+}$ mice at 180 days old of age. The actual (blue circles) and the averaged (red triangles, mean ± SEM) numbers of the terminals in per10 cm dendritic branches of dNGIs from the individual animals (n = 5 mice/group, *p=0.00085, $F_{1.19}$ = 15.36, two-way *ANOVA*). (**c**) Whole-cell currents from individual dNGIs were evoked by the illumination of vChATs at an intensity of 5 mV in slices from AD mice at 150 or 180 days old of age and the age-matched controls. (**d**) The actual (blue circles) and the averaged (red triangles, mean ± SEM) values of the normalized peak amplitude (NPA) of synaptic currents evoked by an intensity of 5 mV. The peak currents were normalized to those evoked by an intensity of 1 mV (defined as 1.0, n = 9 mice/group, *p =0.00075, $F_{1.35}$ = 16.53 two-way *ANOVA* with *Bonferroni* post-hoc test).

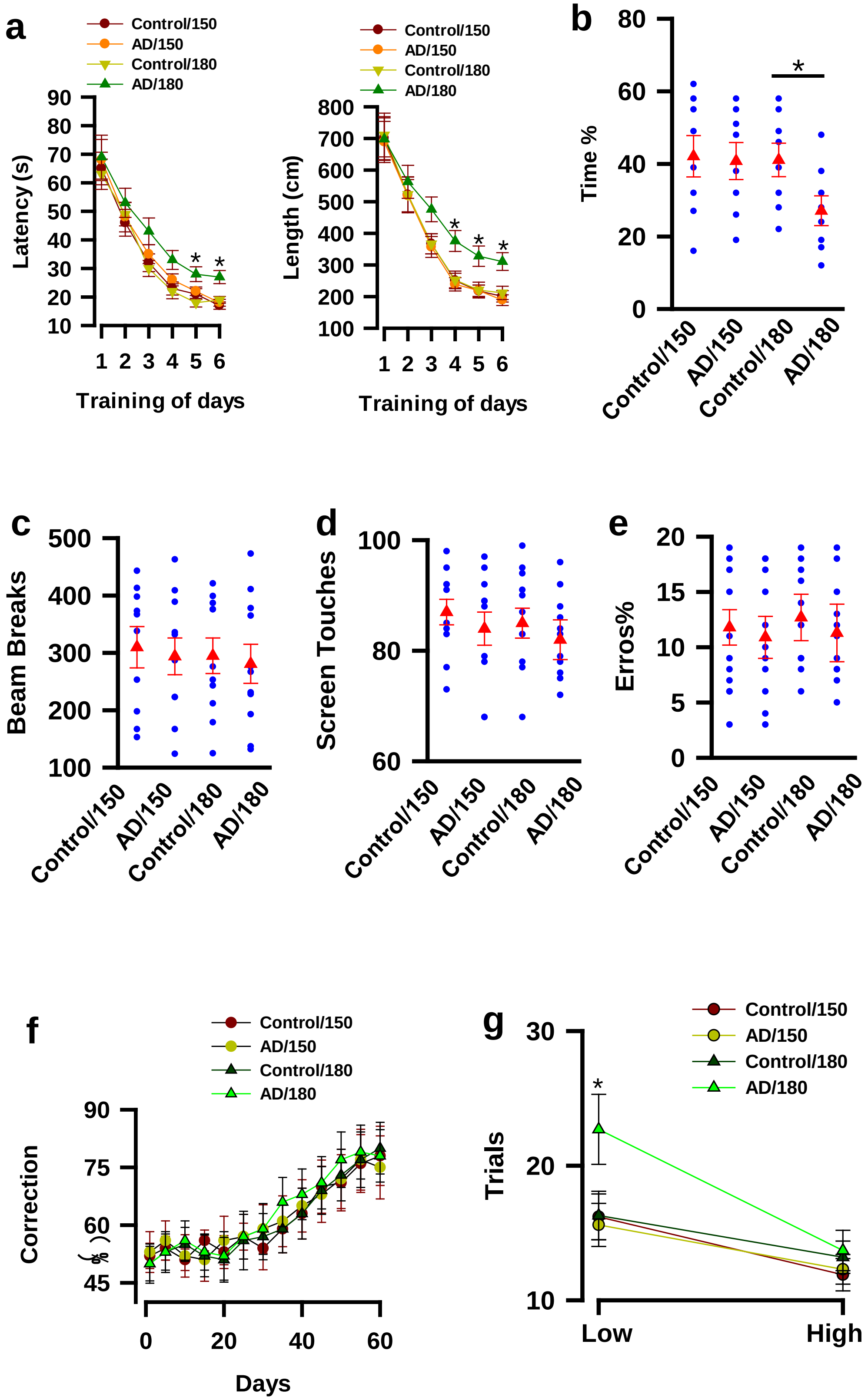
a
Control/150
AD/150
Control/180
AD/180
Latency (s)
90
80
70
60
50
40
30
20
10
1 2 3 4 5 6
Training of days
*
*
Control/150
AD/150
Control/180
AD/180
Length (cm)
800
700
600
500
400
300
200
100
1 2 3 4 5 6
Training of days
*
*
*
b
Time %
80
60
40
20
0
*
Control/150
AD/150
Control/180
AD/180
c
Beam Breaks
500
400
300
200
100
Control/150
AD/150
Control/180
AD/180
d
Screen Touches
100
80
60
Control/150
AD/150
Control/180
AD/180
e
Erros%
20
15
10
5
0
Control/150
AD/150
Control/180
AD/180
f
Control/150
AD/150
Control/180
AD/180
Correction (%)
90
75
60
45
0
20
40
60
Days
g
Control/150
AD/150
Control/180
AD/180
Trials
30
20
10
*
Low
High

Supplementary Fig. 11. **Spatial learning and memory is impaired in AD mice.** (**a, b**) Latencies and swim length (**a**) in searching for a hidden platform in the Morris water maze during the training session and the actual (blues circles) and the averaged (red triangles, mean ± SEM) values of the percentage time spent in a targeting quadrant (**b**) during the probe trials (n = 9 mice/group, $F_{1.35}$ = 13.21, *p = 0.0029, two-way *ANOVA*). (**c-e**) AD mice show normal performance during the pre-training sessions. AD mice at 150 ± 5 or 180 ±5 days old of age and the age-matched controls were pre-trained. The actual (blues circles) and the averaged (red triangles, mean ± SEM) values of beam breaks (c), screen touches (d) and the percentage of incorrect responses (e) are plotted (n = 1 mice/group). **(f)** AD mice show normal PAL performance after the completion of pre-training. Data are mean ± SEM (n = 11 mice/group). **(g)** AD mice show abnormal 2-CSD performance. AD mice at 90 ± 2 days or 120 ± 2 days old of age and the age-matched control mice were conducted with pre-training and followed by PAL. After the completion of PAL, mice were performed with 2-CSD. A plot shows the trials that are required to reach a criterion at low and high separation (mean ± SEM, n = 11 mice/group, $F_{1.43}$ = 11.89, *p = 0.0039, two-way *ANOVA*).

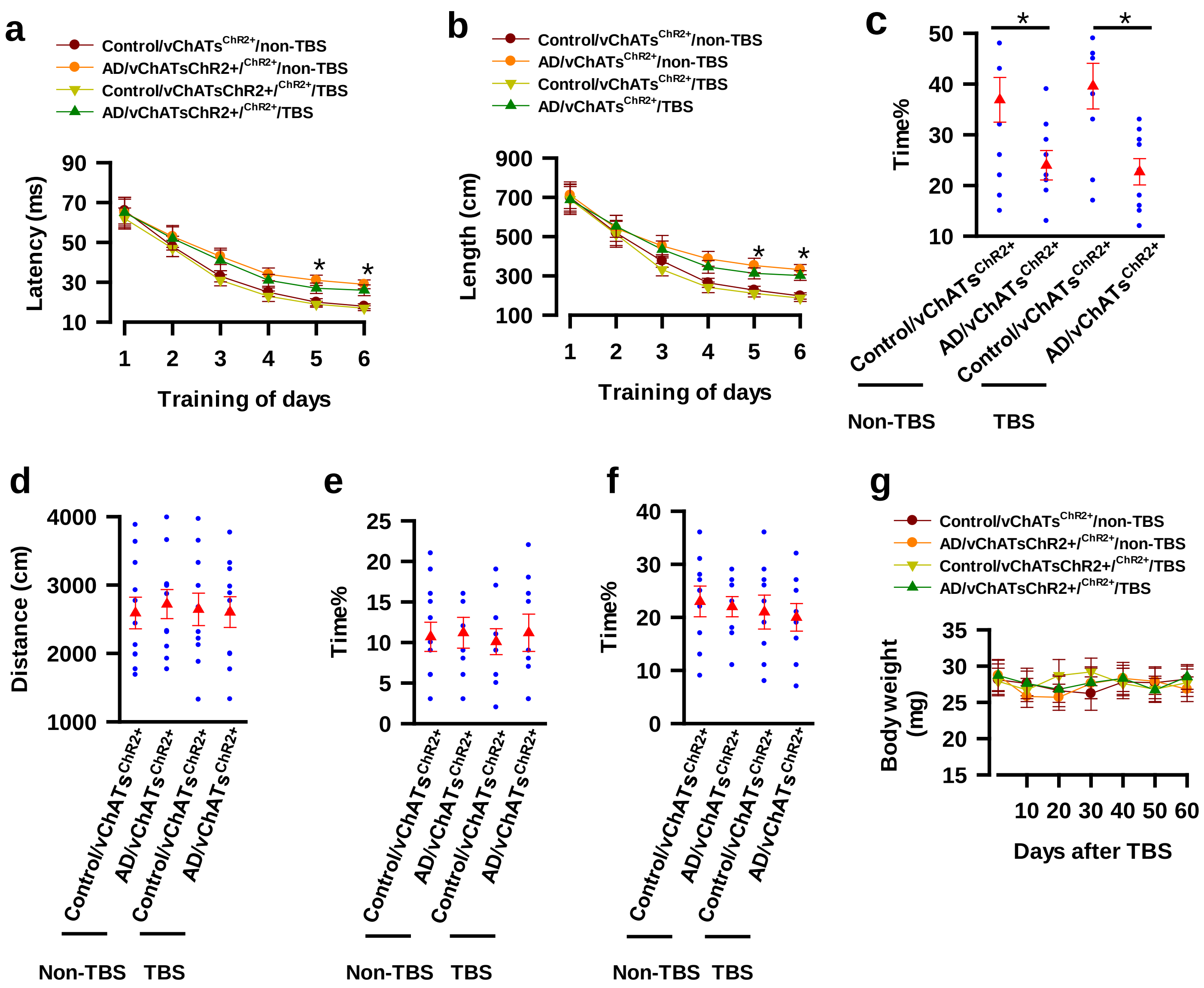

Supplementary Fig. 12. TBS treatment does not affect the performance in the Morris water maze tests. (a-c) Latencies (**a**) and swim length (**b**) in searching for a hidden platform in the maze during the training session and the actual (blue circles) and the averaged (red triangles, mean ± SEM) values of the percentage time spent (**c**) in a targeting quadrant during the probe trials for the individual AD/vChATs^{ChR2+} mice at 180 ± 5 days old of age and the age-matched control/vChATs^{ChR2+} mice treated without (non-TBS) or with TBS. Data are mean ± SEM (n = 9 mice/group, $F_{1.35} = 7.22$, *$p < 0.001$, two-way *ANOVA*). **(d-g)** Distance traveled (**d**) and time spent with the familiar (**e**) and the novel (**f**) objects in the open fields and body weight (**g**) of the AD/vChATs^{ChR2+} mice at 180 ± 5 days old of age and the age-matched control/vChATs^{ChR2+} mice treated without (non-TBS) or with TBS. Data are mean ± SEM (n = 9 mice/group).

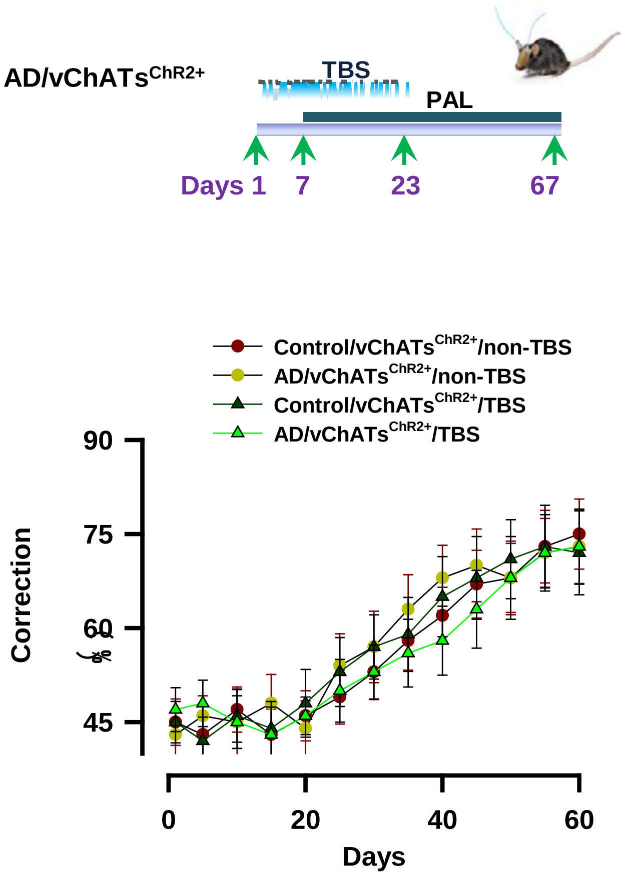

Supplementary Fig. 13. The experimental schedules (top) show that mice at 120 ±2 days old of age were treated with 16-days TBS. 7 days after the beginning of TBS, mice were performed with PAL. A plot show that the percentage of corrections is not affected by TBS (mean ± SEM, n = 11 mice/group).

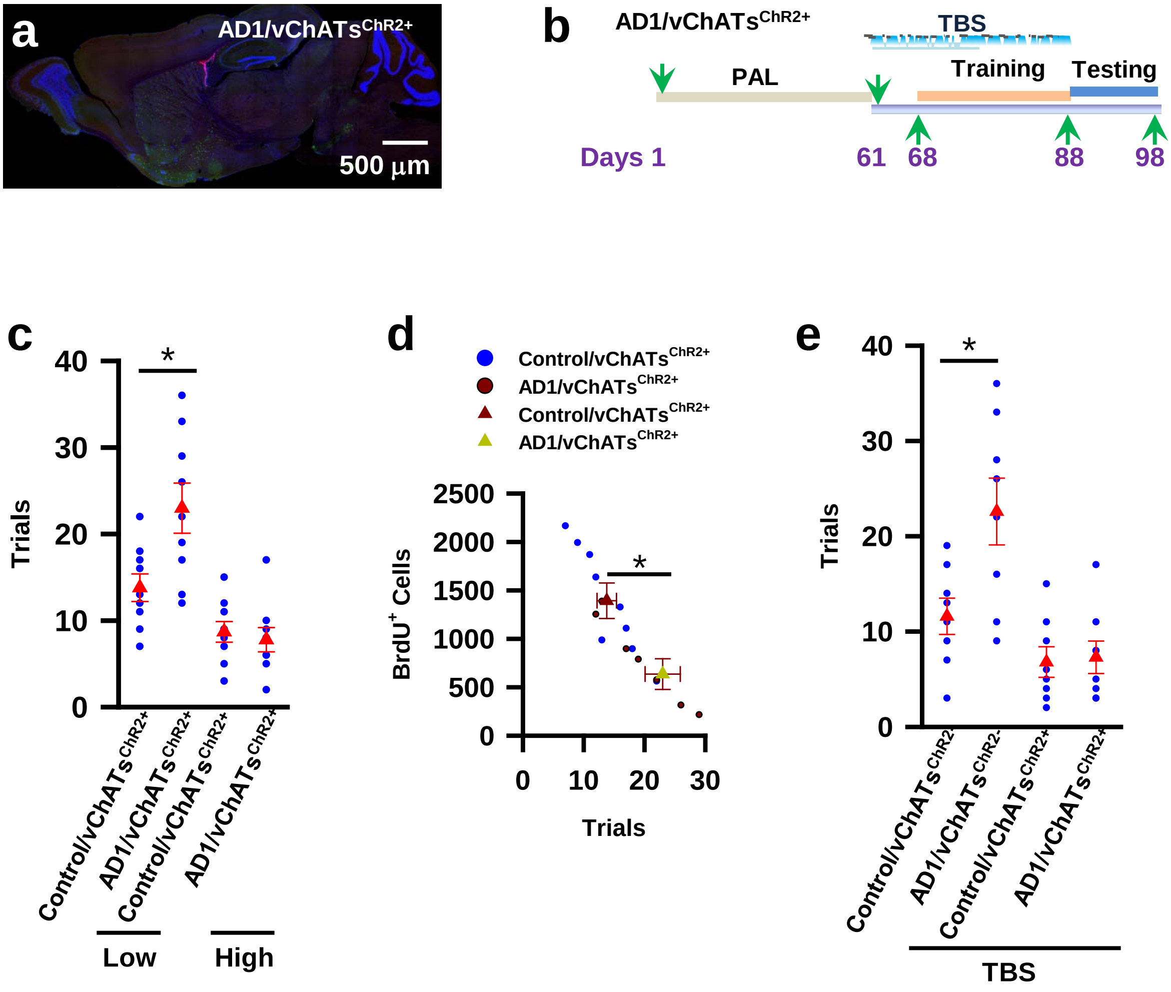

Supplementary Fig. 14. (**a**) A representative image shows a section from AD1/vChATs^{ChR2+} mice that were generated by breeding the AD1 (mutant mice were expressed with APPKM670/671NL(Swedish) and the PSEN1deltaE9 mutant geneses) mice with vChATs^{ChR2+} mice. Control/vChATs^{ChR2+} and AD1/vChATs^{ChR2-} mice from the same litters were used as controls. (**b**) The experimental schedules show that mice at 60 ± 1 days old of age were performed with PAL. 7 days before the completion of PAL, mice were administered with a single dose of BrdU. After the completion of PAL, mice were treated with TBS. 7 days after the beginning of TBS, mice were applied with 2-CSD training and followed by 2-CSD tests. (**c**) The actual (blues circles) and averaged (triangles, mean ± SEM) trials that are required to reach a criterion at a low or high separation are plotted (N = 9 mice/group, $F_{1.35}$ = 29.01, P = 0.0018, two-way *ANOVA*). (**d**) The actual (circles) and averaged (red triangles, mean ± SEM) numbers of BrdU$^+$ cells in the dorsal dentate gyrus are plotted against the actual (circles) and the averaged (red triangles, mean ± SEM) trials that are required to reach a criterion at a low separation of 2-CSD in Supplementary Fig. 13C (n = 9). (**e**) The actual (blues circles) and the averaged (red triangles, mean ± SEM) trials that are required to reach a criterion at low separation are plotted (N = 8 mice/group, $F_{1.31}$ = 8.91, P = 0.0012, two-way *ANOVA*).
